# The Influence of Pregnancy Risk Factors on Patterns of Sensory Processing Disorders and Motor Development

**DOI:** 10.3390/jcm14238429

**Published:** 2025-11-27

**Authors:** Magdalena Szczepara-Fabian, Ewa Emich-Widera, Beata Kazek, Justyna Paprocka

**Affiliations:** 1 Early Intervention Center, Polish Society for People with Intellectual Disability, 44-240 Zory, Poland; 2 Department of Pediatric Neurology, Medical University of Silesia in Katowice, 40-752 Katowice, Poland; eemich-widera@sum.edu.pl (E.E.-W.); jpaprocka@sum.edu.pl (J.P.); 3 Child Development Support Center “Persevere”, 40-583 Katowice, Poland

**Keywords:** sensory processing disorder, risk factors, motor development, patterns of sensory processing disorders

## Abstract

**Background/Objectives:** The objective was to establish whether particular SPD risk factors are correlated with particular SPD patterns and whether these factors affect the motor development of children **Methods:** The study procedures included medical examinations, conducted by a pediatrician/child neurologist, and evaluations, performed by a physiotherapist/sensory integration specialist, which were performed on the study group. **Results:** The study included 156 Caucasian children with SPD aged 3 to 12 years. The results of this study demonstrate that serological conflict shows correlations with taste, smell, and visual hyposensitivity. Fetal heart rate fluctuations, indicated in CTG, are correlated with tactile hypersensitivity and vestibular hypersensitivity, and cesarean delivery is correlated with auditory hyposensitivity. Incubator care is correlated with tactile hypersensitivity and auditory hyposensitivity. Intrauterine infections are correlated with vestibular hypersensitivity. Delayed motor development is correlated with bed rest in the third trimester of pregnancy, intrauterine infections, and incubator care. **Conclusions:** We conclude that children’s development must be monitored scrupulously in particular areas of sensory processing with regard to any of the abovementioned risk factors occurring in affected children. It is important to pay special attention to intrauterine infections, bed regimes in the third trimester of pregnancy, and incubator care, because these factors may have a negative impact on motor development.

## 1. Introduction

Sensory processing disorder (SPD) is an impaired reaction to sensory stimuli coming from the external environment and/or from within our body. As a result, a child’s development becomes disturbed, which leads to difficulties in everyday functioning and achieving new skills [1,2]. According to the epidemiological studies conducted among Western populations, SPD is estimated to occur in about 15–16% of the population in the developmental stage [3,4,5,6,7,8,9,10,11,12]. Knowledge of the etiology and pathomechanisms leading to sensory processing disorder, as well as other neurodevelopmental disorders, has yet to be comprehensive. What has been established is that sensory systems function within neural networks and circuits. A stimuli-conductive synaptic transmission happens via relevant neurotransmitters. Within this process, a large-enough singular or multi-synaptic action potential is created which, in turn, becomes a causative factor of either the activation or blockage of a signal. Damaged neural networks, qualitative and/or quantitative impairments of neurotransmitters, and long-lasting, high-potential stimuli result in incorrect functioning of singular or integrated sensory systems. The physiological development of sensory processing as well as its impairments and corrective processes are directly tied to neuroplasticity. Neuroplasticity is known to be a characteristic of the nervous system that ensures its adaptivity, ability to change, self-regulation, learning, and remembering. It is currently believed that a basic framework of connections within the central nervous system develops during the developmental phase (starting in the fetal stage of development), based on genetic predispositions. It acts as a matrix for building subsequent neural structures, relevant in terms of neurotransmission mechanisms. These progressions are more difficult to change due to new experiences that are connected to physiological processes, known as developmental plasticity. An important factor of the etiopathogenesis of SPD is the occurrence of pregnancy–delivery risk factors, which might negatively affect a child during the fetal, peripartum, and postpartum phases of life.

A great deal of variation in the clinical picture, in terms of the occurrence of particular symptoms, their exacerbation, and their impact on all areas of a child’s development, is observed. There are three main areas in the clinical picture of sensory processing disorder: sensory modulation disorder, sensory discrimination disorder, and sensory-based motor disorder. Sensory-based motor disorder includes posture disorders and dyspraxia. Dyspraxia is a very interesting issue concerning perceptual motor dysfunction that is manifested by the impairment or immaturity of the control of movement. It can affect the areas of gross and fine motor skills, as well as oral motor skills. Within the area of gross motor skills, dyspraxia, in its early phase of development, is manifested, for example, in delayed achievement of developmental milestones and delay in the development of postural control and motor coordination [13].

There are very few publications available regarding a possible connection of one of several specific risk factors to any type of sensory disorder. Additionally, these disorders are treated en bloc. The knowledge of the risk factors can be helpful in the process of the early identification of neurodevelopmental disorders which is crucial for proper therapeutic support of the developmental potential and self-esteem of children. Given the significant prevalence of SPD, and the family and social costs, these issues need to be addressed.

The objective of this study is to establish whether the particular risk factors of SPD are correlated with the individual SPD patterns, including whether the pregnancy and childbirth risk factors affect the shaping of the postural control and the motor development of a child. This project is the continuation of analyses conducted among a group of children with SPD. The first part of the study, which was described in detail in the article Szczepara-Fabian, M., et.al. (2025) [14], established which of the many pregnancy and childbirth risk factors seem to be significant as predictors of the occurrence of SPD.

## 2. Materials and Procedures

### 2.1. Search Strategy

The children who were enrolled in the SPD group were referred by doctors, physiotherapists, clinical speech therapists, and educators. These children presented with developmental problems that resembled SPD. Next, the parents completed the application questionnaires. The inclusion criteria were not met by 3 of the 159 children. The group consisted of 156 children, with suspected SPD, and their biological parents residing in the Silesian region (south-west Poland). In order to select the study group, a complex assessment was conducted. The procedures utilized for this purpose included a medical examination performed by a child neurologist/pediatrician, as well as an examination performed by a physiotherapist/SI specialist. The examination performed by a physiotherapist/SI specialist was conducted in a physiotherapy office using age-appropriate diagnostic tools, such as The Checklist of the American Occupational Therapy Association, Classification DC: 0–5, a case report form, and the Southern California Sensory Integration Test (SCSIT). Besides the analysis of the data gathered with the use of the abovementioned tools, free observation and guided play were significant in the final evaluation.

### 2.2. Eligibility Criteria

The studied group (SPD) in both research studies, described above, consisted of the same 156 children with a diagnosed sensory processing disorder, who live in the Silesia region, as well as their biological parents. The inclusion criteria, besides the diagnosis of SPD (but without additional conditions such as intellectual disability and neurological and genetic diseases), included belonging to the Caucasian race group and an age range between 3 and 12. The exclusion criteria included intellectual disability, neurological diseases (particularly epilepsy and cerebral palsy), and genetic diseases.

### 2.3. Procedures

The researchers studied the relationship between the earlier selected significant SPD risk factors, which were described in detail in the article “Selected variables of the risk of sensory processing disorder” [14], and the different SPD patterns. Based on the conducted studies and analyses, the selected risk factors were the following: infections, serological conflict, stressful events during pregnancy, bed rest in the 2nd trimester, bed rest in the 3rd trimester, fetal heart rate fluctuations indicated in CTG, cesarean delivery, premature birth, low birth weight, incubator care, intrauterine infections, and grade II intraventricular hemorrhage [14]. Special attention was paid to delayed motor development, which is one of the important symptoms of dyspraxia. It was identified from the reverberations of dyspraxia and analyzed as a separate parameter. Motor development delay was declared when the delay in the motor area was at least 2 standard deviations from the mean [15].

### 2.4. Statistical Analysis

The statistical analysis of the obtained results was performed using Excel 2007 and STATISTICA v.12. The result of the statistical analysis were deemed statistically significant if the achieved level of significance *p* was smaller or equal to 0.05.

The sensory patterns were described by numbers and percentage of occurrence. Fisher’s two-tailed exact test was used in the statistical analysis of the differences between the groups of boys and girls.

Multivariate logistic regression analysis was used in order to evaluate which risk factors are interdependent with particular sensory disorders. Due to the large total number of risk factors in comparison to the number of participants in the studied group, it was not possible to analyze them and their interdependence in relation to particular sensory disorder simultaneously; therefore, the analysis in the first phase was conducted by using the Wald test for the following three groups of risk factors: pregnancy (Table S1), perinatal (Table S2) and postnatal (Table S3) factors.

In the second phase of the study, only these risk factors were introduced to the analysis of the correlation with the particular sensory disorder for which the statistically significant result was achieved in the first phase. The significance of the logistic regression model and the odds ratio (ORs) with the 95% confidence leve achieved with the applied model were provided in the summary of the analyses of the second phase.

## 3. Results

Table 1 shows the occurrence of SPD patterns and the parameter of motor development delay in the studied group.

The evaluation of the interdependencies of the pregnancy, childbirth, and postpartum risk factors, which were analyzed by utilizing multivariate logistic regression analysis, are presented in Tables S1–S3.

Table S1 shows the evaluation of interdependencies of pregnancy risk factors, sensory processing disorder, and motor development delay (Wald test results). Serological conflict shows correlation with taste, smell, and visual hyposensitivity. Bed rest in the third trimester of pregnancy is correlated with motor development delay.

Table S2 shows the evaluation of interdependencies between perinatal risk factors, sensory processing disorder patterns, and the motor development delay parameter (Wald test results). Fetal heart rate fluctuations in CTG shows correlation with tactile hypersensitivity and vestibular hyposensitivity. Cesarian delivery shows correlation with auditory hyposensitivity.

Table S3 shows the evaluation of interdependency between postnatal risk factors and intrauterine infection and between sensory processing disorder and the delayed motor development parameter (Wald test results). Incubator care is correlated with tactile hypersensitivity and auditory hypersensitivity. Additionally, incubator care is correlated with motor development delay. Intrauterine infection and vestibular hypersensitivity are interdependent. Additionally, intrauterine infection is correlated with motor development delay.

Table 2 is a summary of Tables S1–S3.

Table 3 shows the values of the odds ratios (ORs). The “delayed motor development” parameter displays interdependency with a bed regime in the third trimester (OR of approx. 3.5), intrauterine infection (with high-value OR: 12), and incubator care (OR of approx. 3.5), with *p* < 0.00001. Interdependency is also indicated between serological conflict, taste hyposensitivity, smell hyposensitivity, and visual hyposensitivity (all ORs of approx. 4.5).

## 4. Comments and Discussion

The objective of our study was to establish whether particular SPD risk factors are correlated with particular SPD patterns and whether these factors affect the motor development of children. One hundred and fifty-six Caucasian children with SPD between the ages of 3 and 12 years old took part in this research study. Through this study, we concluded that serological conflict shows correlations with taste, smell, and visual hyposensitivity. Fetal heart rate fluctuations indicated in CTG are correlated with tactile hypersensitivity and vestibular hypersensitivity, and cesarean delivery is correlated with auditory hyposensitivity. Incubator care is correlated with tactile hypersensitivity and auditory hyposensitivity. Intrauterine infections are correlated with vestibular hypersensitivity. Delayed motor development is correlated with bed rest in the third trimester of pregnancy, intrauterine infections, and incubator care.

The infants, whose mothers experienced bed regimes during pregnancy, displayed symptoms of apathy, reluctance to suck, and poor repertoire of general movements (GMs). We hypothesize that this could be a result of internal sensory deprivation within the somatosensory areas (touch, proprioception, and vestibular systems). Efficient functioning of these senses is fundamental for the development of the subsequent achievements in perception and optimal reaction to environmental signals. Somatosensory senses develop in the very early phase of fetal life and for that reason they are very sensitive to damaging factors. The earliest reaction of the vestibular system (commonly called the labyrinth) is the Moro reflex, which can be observed as early as starting from the 9th week of pregnancy [16]. On the other hand, the deep sensory receptors, otherwise referred to as proprioception, are stimulated during the movements of the fetus. The embryo starts moving around the 7th week of pregnancy, when spontaneous general movements (GMs), which are an integral part of the typical development of human beings, start to generate endogenously [17]. Around week 17 of pregnancy, the fetus living in the intrauterine environment is mainly stimulated by its own movements and the movements of its mother [18]. In order to intensify the reception of tactile stimuli, lanugo grows on the entire body of the fetus to later disappear, starting from week 33 of pregnancy. Every hair of lanugo is equipped with touch receptors and even the smallest bending of the hair results in stimulation of the tactile sense [18]. Poor integration of somatosensory sensations might cause difficulties with achieving and/or retaining the optimal level of stimulation and a slower development of postural reactions, including oculomotor reactions. This happens this way because the motor abilities of human beings depend on, among other things, afferent sensory information. Hickok, on the other hand, emphasizes that “Somatosensory feedback information is a critical element of movement control.” [19].

According to Estes et al., maternal immune activation (MIA) can be caused by a pathogenic infection in the mother, and it can cause the development of neurodevelopmental disorders [20]. As far as neurodevelopmental disorders are concerned, it is believed that induction requires exposure to more than one risk factor, and MIA often starts a cascade of adverse factors [21]. This way, the individual becomes more prone to the consequences of genetic mutations and environmental exposition and present disease symptoms in later stages of development [22].

Physiologic birth plays an important role in the development of a child’s somatosensory senses. Cesarian delivery could impede the natural training of consolidating the tactile, proprioceptive, and vestibular senses [23]. Efficient functioning of these senses is fundamental for developing perception initiated from the area of the auditory and visual senses [24]. We have also just recently learned that the path of birth could affect the level of development of the smell sense at birth. It appears as though the noradrenaline that is released during childbirth in the organism of the fetus has a positive effect on the optimal sensory reaction within the area of smell after birth [25].

Newborns and infants that are under incubator care require special attention as they have direct contact with life-saving medical equipment. The infants’ undeveloped skin is exposed to, among other things, pressure and medical injuries after attaching sensors or after contact with the disinfectants of medical personnel [26,27]. The time spent in incubators in intensive care units also exposes the infants to many additional non-physiological auditory stimuli and, at the same time, decreases their opportunity to experience oral stimulation [28]. We can assume with high probability that the exposure to irritating tactile stimuli and the poor vestibular and auditory stimulation at such an early phase of development constitute a biological base for the occurrence of sensory processing disorders in the later stages of life.

Serological conflict is a novelty as an SPD risk factor in the literature on this topic. The evidenced correlation between serological conflict and disturbed modality in the visual, taste, and smell areas is intriguing and worth conducting further clinical investigation on. The disturbed modality in the areas of these senses prevents the creation of a consolidated sensory experience during breastfeeding and may lead to difficulties in food intake. The literature more often provides expressions such as “sensory food aversion” and “avoidant food intake” due to the occurrence of aversive reactions to sensory characteristics of food, such as smell, taste, appearance, texture, and even temperature of food products [29]. Eating disorders become, more often, a clinical problem in the sense of a broadly understood early intervention.

### 4.1. Limitations of the Study

The sample consists exclusively of Caucasian children from a single region (Silesia, Poland) with strict exclusion of comorbidities. SPD often co-occurs with developmental and neurological conditions, and findings may not generalize across populations. There is a need for longitudinal, multicenter studies to validate these associations.

The authors conclude that it would be optimal to perform long-term observations within large, more diverse, random cohorts of pregnant women and their children in the future and monitor them from birth to pre-school age.

### 4.2. Future Research Directions

Future research directions include replication in larger, more diverse cohorts, use of longitudinal designs, and integration of neurobiological measures.

## 5. Conclusions

It is important to pay attention to the correlation between auditory hyposensitivity and cesarian delivery and incubator care and the correlation between serological conflict and visual, taste and smell hyposensitivity. The disturbed modality in these areas makes it difficult for the creation of an integrated sensory experience and has a negative impact on children’s psychomotor development. This is why it is very important to introduce the kind of care and nursing activities that are adequate for the perceptive, cognitive, and motor abilities of children and prevent any further reverberations of SPD this way.

From the point of view of the physioprophylaxis of developmental disorders, there is a high probability that the development of movement control in infants with a history of pregnancy/childbirth risk factors (intrauterine infection, bed regime in the third trimester, incubator care) will be disturbed and, consequently, cause delays in motor development. This is the reason why it is important to consider including physiotherapeutic exams in the standard ambulatory postnatal care of infants.

It is recommended that developmental aid, considering each child’s individual needs, is implemented early. It is vital that caregivers and professionals perform comprehensive evaluation aimed at establishing sensitivity levels (which areas are hypersensitive and which are hyposensitive). This would be the basis for adopting stimulation practices optimized in terms of frequency and intensity so that overstimulation or understimulation can be avoided.

## Figures and Tables

**Table 1:** SPD patterns and the delayed motor development parameter occurring among children in the study group depending on gender.

SPD Pattern and Additional Studied Parameter	Gender		Fisher’s Exact Test
	Male (*n* = 110; 100%)	Female (*n* = 46; 100%)	
Tactile hypersensitivity	38 (34.6%)	21 (45.7%)	NS (*p* = 0.21)
Tactile hyposensitivity	40 (36.4%)	19 (41.3%)	NS (*p* = 0.59)
Proprioceptive hypersensitivity	0 (0%)	0 (0%)	---
Proprioceptive hyposensitivity	84 (76.4%)	35 (76.1%)	NS (*p* = 0.56)
Vestibular hypersensitivity	13 (11.8%)	6 (13.0%)	NS (*p* = 0.51)
Vestibular hyposensitivity	75 (68.2%)	30 (65.2%)	NS (*p* = 0.71)
Sensory seeking	38 (34.6%)	16 (34.8%)	NS (*p* = 0.99)
Taste hypersensitivity	6 (5.5%)	7 (15.2%)	NS (*p* = 0.06)
Taste hyposensitivity	13 (11.8%)	7 (15.2%)	NS (*p* = 0.61)
Smell hypersensitivity	6 (5.5%)	6 (13.0%)	NS (*p* = 0.18)
Smell hyposensitivity	14 (12.7%)	6 (13.0%)	NS (*p* = 0.99)
Auditory hypersensitivity	11 (10.0%)	3 (6.5%)	NS (*p* = 0.76)
Auditory hyposensitivity	20 (18.2%)	5 (10.9%)	NS (*p* = 0.34)
Visual hypersensitivity	6 (5.5%)	3 (6.5%)	NS (*p* = 0.72)
Visual hyposensitivity	11 (10.0%)	1 (2.2%)	NS (*p* = 0.11)
Dyspraxia */	14 of 9 (15.7%)	0 of 31 (0.0%)	*p* = 0.02
Postural disorders */	49 of 89 (55.1%)	13 of 30 (43.3%)	NS (*p* = 0.30)
Delayed motor development	60 (54.6%)	29 (63.0%)	NS (*p* = 0.38)

Attention: */—the results are not available for all children.

**Table 2:** Summary: Evaluation of interdependencies between risk factors, sensory processing disorder patterns, and motor development delay parameter (Wald test results).

SPD Patterns and Additional Studied Parameter	Risk Factors	Evaluation of Interdependencies
Tactile hypersensitivity	Fetal Heart Rate Fluctuations in CTG	*p* = 0.04
	Incubator Care	*p* = 0.02
Vestibular hypersensitivity	Fetal Heart Rate Fluctuations in CTG	*p* = 0.002
	Intrauterine Infection	*p* = 0.007
Auditory hyposensitivity	Incubator Care	*p* = 0.05
	Cesarian Delivery	*p* = 0.02
Visual hyposensitivity	Serological Conflict	*p* = 0.02
Taste hyposensitivity	Serological Conflict	*p* = 0.006
Smell hyposensitivity	Serological Conflict	*p* = 0.004
Delayed motor development	Bed Rest in 3rd Trimester	*p* = 0.03
	Incubator Care	*p* = 0.05
	Intrauterine Infection	*p* = 0.03

**Table 3:** Odds ratio (OR) values and their 95% confidence intervals (95% CIs) achieved in the logistic regression analysis of the significant interdependencies between the occurrence of risk factors, sensory processing disorder, and the delayed motor development parameter.

SPD Patterns and Additional Parameter	Model Significance	Risk Factors					
		Serological Conflict	Bed Regime in 3rd Trimester	Fetal Heart Rate Fluctuations in CTG	Cesarian Delivery	Incubator Care	Intrauterine Infection
Tactile hypersensitivity	*p* = 0.01	---	---	OR = 2.36 (1.05; 5.33)	---	OR = 2.03 (0.94; 4.37)	---
Tactile hyposensitivity	---	---	---	---	---	---	---
Proprioceptive hyposensitivity	---	---	---	---	---	---	---
Vestibular hypersensitivity	*p* = 0.0006	---	---	OR = 4.36 (1.48; 12.8)	---	---	OR = 3.48 (0.98; 12.4)
Vestibular hyposensitivity	---	---	---	---	---	---	---
Sensory seeker	---	---	---	---	---	---	---
Taste hypersensitivity	---	---	---	---	---	---	---
Taste hyposensitivity	*p* = 0.02	OR = 4.43 (1.42; 13.8)	---	---	---	---	---
Smell hypersensitivity	---	---	---	---	---	---	---
Smell hyposensitivity	*p* = 0.01	OR = 4.43 (1.42; 13.8)	---	---	---	---	---
Auditory hypersensitivity	---	---	---	---	---	---	---
Auditory hyposensitivity	*p* = 0.03	---	---	---	OR = 2.86 (1.05; 7.81)	OR = 1.58 (0.81; 4.10)	---
Visual hypersensitivity	---	---	---	---	---	---	---
Visual hyposensitivity	*p* = 0.03	OR = 4.64 (1.23; 17.6)	---	---	---	---	---
Dyspraxia	---	---	---	---	---	---	---
Postural disorders	---	---	---	---	---	---	---
Delayed motor development	*p* < 0.00001	---	OR = 3.57 (1.38; 9.25)	---	---	OR = 3.52 (1.36; 9.08)	OR = 12.0 (1.46; 99.0)

## Data Availability

The data that support the findings of this study are available and can be obtained from the corresponding author, upon reasonable request.

## Supplementary Materials

The following supporting information can be downloaded at https://www.mdpi.com/article/10.3390/jcm14238429/s1, Table S1: Evaluation of interdependencies of pregnancy risk factors, sensory processing disorder, and motor development delay (Wald test results); Table S2: Evaluation of interdependencies between perinatal risk factors, sensory processing disorder patterns, and motor development delay parameter (Wald test results); Table S3: Evaluation of interdependency between postnatal risk factors in relation to intrauterine infection and sensory processing disorder in relation to delayed motor development parameter (Wald test results).

## Abbreviation

The following abbreviations is used in this manuscript:

SPD	Sensory Processing Disorder
