# Supplementary material for: The Influence of Pregnancy Risk Factors on Patterns of Sensory Processing Disorders and Motor Development
Source: J Clin Med. 2025 Nov 27;14(23):8429. doi: 10.3390/jcm14238429 (PMC12693627; doi:10.3390/jcm14238429)
Supplement: Supplementary file 1 [file jcm-14-08429-s001.zip › jcm-3877352-supplementary.pdf]

Table S1. Evaluation of interdependencies of pregnancy risk factors, sensory processing disorder, and motor development delay (Wald test results)

SPD Pattern and Additional Studied Parameter	Model Significance	Risk Factors			
		Serological Conflict	Stressful Events During Pregnancy	Bed Rest in 2nd Trimester	Bed Rest in 3rd Trimester
Tactile hypersensitivity	NS (p=0.93)	NS (p=0.66)	NS (p=0.80)	NS (p=0.99)	NS (p=0.54)
Tactile hyposensitivity	NS (p=0.72)	NS (p=0.27)	NS (p=0.50)	NS (p=0.55)	NS (p=0.99)
Proprioceptive hyposensitivity	NS (p=0.92)	NS (p=0.69)	NS (p=0.69)	NS (p=0.41)	NS (p=0.54)
Vestibular hypersensitivity	NS (p=0.99)	NS (p=0.95)	NS (p=0.64)	NS (p=0.72)	NS (p=0.84)
Vestibular hyposensitivity	NS (p=0.87)	NS (p=0.95)	NS (p=0.57)	NS (p=0.53)	NS (p=0.99)
Sensory seeking	NS (p=0.49)	NS (p=0.70)	NS (p=0.87)	NS (p=0.10)	NS (p=0.48)
Taste hypersensitivity	NS (p=0.47)	NS (p=0.54)	NS (p=0.08)	NS (p=0.67)	NS (p=0.68)
Taste hyposensitivity	p=0.007	p=0.006	NS (p=0.31)	NS (p=0.43)	NS (p=0.82)
Smell hypersensitivity	NS (p=0.37)	NS (p=0.45)	NS (p=0.06)	NS (p=0.71)	NS (p=0.71)
Smell hyposensitivity	p=0.04	p=0.004	NS (p=0.31)	NS (p=0.12)	NS (p=0.83)
Auditory hypersensitivity	NS (p=0.73)	NS (p=0.67)	NS (p=0.18)	NS (p=0.98)	NS (p=0.95)
Auditory hyposensitivity	NS (p=0.92)	NS (p=0.51)	NS (p=0.62)	NS (p=0.97)	NS (p=0.66)
Visual hypersensitivity	NS (p=0.88)	NS (p=0.92)	NS (p=0.63)	NS (p=0.84)	NS (p=0.49)
Visual hyposensitivity	p=0.05	p=0.02	NS (p=0.84)	NS (p=0.12)	NS (p=0.56)
Dyspraxia	NS (p=0.22)	--- /*	NS (p=0.13)	NS (p=0.29)	NS (p=0.56)
Postural disorders	NS (p=0.92)	NS (p=0.82)	NS (p=0.46)	NS (p=0.99)	NS (p=0.62)
Delayed motor development	p=0.04	NS (p=0.18)	NS (p=0.58)	NS (p=0.79)	p=0.03

Attention: /* Serological conflict was removed from the analysis as none of the children with serological conflict had dyspraxia.

Table S2. Evaluation of interdependencies between perinatal risk factors, sensory processing disorder patterns, and the motor development delay parameter (Wald test results).

SPD Pattern and Additional Studied Parameter	Model Significance	Risk Factors			
		Fetal Heart Rate Fluctuations in CTG	Cesarian Delivery	Weeks of Pregnancy HBD<38	Burth Weight <2500 G
Tactile hypersensitivity	p=0.002	p=0.04	NS (p=0.32)	NS (p=0.14)	NS (p=0.30)
Tactile hyposensitivity	NS (p=0.22)	NS (p=0.16)	NS (p=0.32)	NS (p=0.29)	NS (p=0.75)
Proprioceptive hyposensitivity	NS (p=0.18)	NS (p=0.10)	NS (p=0.60)	NS (p=0.91)	NS (p=0.12)
Vestibular hypersensitivity	p=0.002	p=0.002	NS (p=0.09)	NS (p=0.50)	NS (p=0.53)

Vestibular hyposensitivity	NS (p=0.10)	NS (p=0.06)	NS (p=0.87)	NS (p=0.87)	ND (p=0.22)
Sensory seeking	NS (p=0.50)	NS (p=0.23)	NS (p=0.98)	NS (p=0.66)	NS (p=0.51)
Taste hypersensitivity	NS (p=0.68)	NS (p=0.75)	NS (p=0.16)	NS (p=0.81)	NS (p=0.62)
Taste hyposensitivity	NS (p=0.23)	NS (p=0.11)	NS (p=0.93)	NS (p=0.49)	NS (p=0.26)
Smell hypersensitivity	NS (p=0.45)	NS (p=0.78)	NS (p=0.09)	NS (p=0.68)	NS (p=0.55)
Smell hyposensitivity	NS (p=0.48)	NS (p=0.24)	NS (p=0.59)	NS (p=0.43)	NS (p=0.27)
Auditory hypersensitivity	NS (p=0.90)	NS (p=0.80)	NS (p=0.40)	NS (p=0.71)	NS (p=0.94)
Auditory hyposensitivity	p=0.05	NS (p=0.80)	p=0.02	NS (p=0.89)	NS (p=0.47)
Visual hypersensitivity	NS (p=0.46)	NS (p=0.41)	NS (p=0.26)	NS (p=0.92)	NS (p=0.50)
Visual hyposensitivity	NS (p=0.87)	NS (p=0.71)	NS (p=0.91)	NS (p=0.71)	NS (p=0.38)
Dyspraxia	NS (p=0.50)	NS (p=0.23)	NS (p=0.33)	NS (p=0.40)	NS (p=0.50)
Postural disorders	NS (p=0.09)	NS (p=0.08)	NS (p=0.83)	NS (p=0.14)	NS (p0.11)
Delayed motor development	NS (p=0.07)	NS (p=0.37)	NS (p=0.72)	NS (p=0.32)	NS (p=0.17)

Table S3. Evaluation of interdependency between postnatal risk factors and intrauterine infection and between sensory processing disorder and the delayed motor development parameter (Wald test results).

SPD Pattern and Additional Studied Parameter	Model Significance	Risk Factors		
		Incubator Care	Intrauterine Infection	IVH Grade 2
Tactile hypersensitivity	p=0.05	p=0.02	NS (p=0.59)	NS (p=0.16)
Tactile hyposensitivity	NS (p=0.60)	NS (p=0.29)	NS (p=0.50)	NS (p=0.28)
Proprioceptive hyposensitivity	NS (p=0.17)	NS (p=0.07)	NS (p=0.35)	NS (p=0.26)
Vestibular hypersensitivity	p=0.04	NS (p=0.49)	p=0.007	NS (p=0.66)
Vestibular hyposensitivity	NS (p=0.13)	NS (p=0.19)	NS (p=0.18)	NS (p=0.10)
Sensory seeking	NS (p=0.22)	NS (p=0.07)	NS (p=0.82)	NS (p=0.83)
Taste hypersensitivity	NS (p=0.97)	NS (p=0.99)	--- /*	NS (p=0.83)
Taste hyposensitivity	NS (p=0.59)	NS (p=0.61)	NS (p=0.34)	NS (p=0.52)
Smell hypersensitivity	NS (p=0.79)	NS (p=0.53)	--- /#	NS (p=0.88)
Smell hyposensitivity	NS (p=0.66)	NS (p=0.37)	NS (p=0.34)	NS (p=0.83)
Auditory hypersensitivity	NS (p=0.19)	NS (p=0.14)	--- /$	NS (p=0.62)
Auditory hyposensitivity	p=0.05	p=0.05	NS (p=0.45)	NS (p=0.36)
Visual hypersensitivity	NS (p=0.27)	NS (p=0.39)	NS (p=0.30)	NS (p=0.99)
Visual hyposensitivity	NS (p=0.67)	NS (p=0.67)	--- /&	NS (p=0.59)
Dyspraxia	NS (p=0.76)	NS (p=0.33)	NS (p=0.62)	NS (p=0.44)
Postural disorders	NS (p=0.08)	NS (p=0.41)	NS (p=0.07)	NS (p=0.73)

Delayed motor development	p=0.00008	p=0.05	p=0.03	NS (p=0.57)

Attention:
/* - Intrauterine infection was removed from the analysis as none of the children with the infection had taste hypersensivity.
/# - Intrauterine infection was removed from the analysis as none of the children with the infection had smell hypersensivity.
/$ - Intrauterine infection was removed from the analysis as none of the children with the infection had auditory hypersensivity.
/& - Intrauterine infection was removed from the analysis as none of the children with the infection had visual hyposensivity.
